# Distribution of small bowel involvement and its association with clinical outcomes in patients with Crohn’s disease

**DOI:** 10.1097/MD.0000000000035040

**Published:** 2023-10-06

**Authors:** Jin Park, Hae Young Kim, Yoon Jin Lee, Hyuk Yoon, Cheol Min Shin, Young Soo Park, Nayoung Kim, Dong Ho Lee

**Affiliations:** a Department of Internal medicine, Seoul National University Bundang Hospital, Seongnam, South Korea; b Department of Radiology, Asan Medical Center, Seoul, South Korea; c Department of Radiology, Seoul National University Bundang Hospital, Seongnam, South Korea; d Department of Internal Medicine and Liver Research Institute, Seoul National University College of Medicine, Seoul, South Korea.

**Keywords:** Crohn disease, inflammatory bowel diseases, small bowel involvement, tomography, X-ray computed

## Abstract

We aimed to evaluate the distribution of small-bowel involvement in Crohn’s disease (CD) and its association with clinical outcomes. This study included CD patients who underwent computed tomography (CT) at initial diagnosis from June 2006 to April 2021. Two abdominal radiologists reviewed the CT images, and independently rated the presence of “bowel wall thickening,” “stricture,” and “fistula or abscess” in the small bowel segments of jejunum, distal jejunum/proximal ileum, distal ileum, and terminal ileum, respectively. Based on findings of the image review, each patient’s “disease-extent imaging score” and “behavior-weighted imaging score” (a higher score indicative of more structuring or penetrating disease) were calculated. Major clinical outcomes (emergency department [ED] visit, operation, and use of corticosteroids or biologics) were compared according to the 2 scores and L4 involvement by the Montreal classification. The proportions of involvement in the jejunum, distal jejunum/proximal ileum, distal ileum, and terminal ileum were 2.0%, 30.3%, 82.2%, and 71.7%, respectively, identifying 30.3% of patients as having L4 disease and 69.7% of patients as having involvement of multiple segments. Clinical outcomes were not significantly associated with the disease-extent imaging score or L4 involvement. However, significant differences were noted for the ED visits and the use of biologics, according to the behavior-weighted imaging score. Moreover, in multivariable analysis, disease behavior was the only factor associated with all clinical outcomes (ED visit, hazard ratio [HR] 2.127 [1.356–3.337], *P *= .001; operation, HR 8.216 [2.629–25.683], *P* < .001; use of corticosteroid, HR 1.816 [1.249–2.642], *P *= .002; and use of biologics, HR 2.352 [1.492–3.708], *P *< .001). Initial disease behavior seems to be a more critical factor for clinical outcomes of CD than the extent or distribution of small-bowel involvement on CT.

## 1. Introduction

Crohn’s disease (CD) is an inflammatory bowel disease that can affect any part of the gastrointestinal tract.^[1]^ It is known that about 80% of Asian patients, and up to 90% of Korean patients with CD have small bowel involvement.^[2,3]^ According to the Asia-Pacific Crohn’s and Colitis Epidemiology Study, 31% of Asian patients with CD had small bowel disease, 24% had colonic disease, 45% had ileocolonic disease, and 5% had upper gastrointestinal disease.^[3]^

There are several diagnostic modalities for evaluating small bowel involvement in CD, including enteroscopy, capsule endoscopy, small bowel follow-through, computed tomography (CT), and magnetic resonance imaging (MRI). Enteroscopy is invasive, and capsule endoscopy cannot be performed in patients with stenosis.^[4]^ Endoscopic evaluation of small bowel involvement is limited. Thus, the role of noninvasive imaging modalities, such as CT or MRI, is very important in CD.^[5–9]^ Due to its relatively higher accessibility compared to MRI, and its excellent performance in evaluating the disease extent, activity, and complications, CT has emerged as an important modality complementing endoscopy for the diagnosis of CD with small bowel involvement.

The Montreal classification is widely used for the classification of CD.^[10]^ The location is divided into L1 (ileal), L2 (colonic), L3 (ileocolonic), and L4 (isolated upper-disease modifier). The behavior is divided into B1 (non-structuring, non-penetrating), B2 (structuring), and B3 (penetrating).^[10]^ The association between L4 disease and clinical outcomes is still elusive with varying results among previous studies. While a few studies suggested that involvement of the upper gastrointestinal tract is associated with worse clinical outcomes, other studies have suggested otherwise.^[11–15]^ Moreover, few studies have investigated the association between the initial disease extent or severity in the small bowel and clinical outcomes in CD. Most previous studies on small bowel involvement in CD focused on the performance of diagnostic modalities such as imaging tests or capsule endoscopy.^[16–18]^ One study investigated the distribution of small bowel involvement, but not its association with clinical outcomes.^[19]^

Thus, we aimed to evaluate the distribution of small bowel involvement in CD through a detailed CT image review, and thereby analyze the association of the disease extent, location, and behavior in the small bowel with clinical outcomes.

## 2. Materials and methods

This study was approved by the Institutional Review Board of the Seoul National University Bundang Hospital (IRB no.: B-2111-723-103). Because this was a retrospective study, the requirement for informed consent was waived by the Institutional Review Board of the Seoul National University Hospital.

### 2.1. Patients

Among the patients with CD who had been enrolled in the IBD cohort of Seoul National University Bundang Hospital, 155 patients who underwent CT at initial diagnosis from June 2006 to April 2021 were identified. Of these patients, 3 patients with disease involvement limited to the colon were excluded. Finally, 152 patients were included in this study.

### 2.2. Study plan

We aimed to evaluate the distribution of small bowel involvement in CD through detailed CT image review, and thereby analyze the association of disease extent, location, and behavior in the small bowel with clinical outcomes. The clinical outcomes analyzed were as follows: the first event since initial diagnosis, emergency department (ED) visit, operation for symptomatic small bowel involvement, new use of corticosteroids, and new use of biologics.

### 2.3. Diagnostic workup

We typically performed colonoscopy and CT of the abdomen and pelvis for the initial diagnostic workup of CD. Most patients underwent CT in our center, while a small number of patients underwent CT elsewhere, the data of which were then registered to our picture archiving and communication system.

### 2.4. Image review of CT and imaging scores according to the disease extent and behavior

Two abdominal radiologists (with 7 and 15 years of experience, respectively), who were blinded to clinical information, reviewed the CT images of all patients retrospectively. The small bowel, excluding the duodenum, was divided into 4 segments. The terminal ileum was defined as the ileum up to 10 cm from the ileocecal valve. The remaining small bowel was divided into the jejunum, distal jejunum/proximal ileum, and distal ileum. The distal jejunum and proximal ileum were combined as 1 segment, because the two could not be differentiated reliably on CT. The radiologists independently rated the presence of “bowel wall thickening,” “stricture,” and “fistula or abscess” in each segment. For the analysis, a lesion was considered to be present only if both radiologists concurred. We defined “L4 by imaging” as the involvement of the distal jejunum/proximal ileum and jejunum on CT images.

First, to analyze clinical outcomes according to disease extent, a score of either 0 or 1 was assigned to each segment, where a score of 1 indicated the presence of any lesion in that segment. We divided the patients into 3 groups based on the total score summed across the small bowel segments (hereafter called the disease-extent imaging score) as follows: 0, 1–2, and 3–4.

Second, to analyze clinical outcomes considering both extent of small bowel and disease behavior, we applied an alternative scoring system, where a score of 0 was assigned for no lesion, 1 for bowel wall thickening, 2 for stricture, 3 for fistula, and 4 for abscess. Thus, 10 was the highest score possible in each segment, with a total score of 40 for all 4 segments (hereafter called the behavior-weighted imaging score). Patients were divided into 3 groups based on the patient distribution across the scores from 0 to 40 as follows: 0–1, 3–4, and ≥5. Patients with a score of 0 or 1 had no lesions or localized bowel wall thickening without stricture or fistula. Patients with a score of 5 or higher had multiple lesions, with at least 1 stricture or fistula.

### 2.5. Statistical analysis

We analyzed the cumulative incidence of the clinical outcomes according to the disease-extent imaging score and location (L4 vs non-L4 disease) using Kaplan–Meier survival analysis and log-rank tests. We performed Cox proportional hazard analyses to identify the risk factors associated with poor clinical outcomes in patients with CD. The analyzed risk factors were age, behavior, location by Montreal classification, L4 by imaging, sex, perianal disease, smoking, extraintestinal manifestation, and disease-extent imaging score.

We had initially planned the analysis based on the disease-extent imaging score and location (L4 disease) without consideration of the behavior-weighted imaging score. However, based on the results of the Cox proportional hazard analyses where disease behavior was the only factor associated with all analyzed clinical outcomes, we added ad-hoc analyses using the behavior-weighted imaging score. As in the pre-planned analyses, cumulative incidence of the clinical outcomes according to the behavior-weighted imaging score was analyzed, and Cox proportional hazard analyses were performed.

Variables with a *P* value of <.1 in univariable analysis were subjected to multivariable Cox proportional hazards analysis. Statistical significance was set at *P* value <.05. We also calculated Cohen’s kappa coefficients for inter-observer agreement between the 2 radiologists in the retrospective CT image review. All statistical analyses were conducted using the statistical software SPSS for Windows (version 25.0; IBM Corp., Armonk, NY).

## 3. Results

### 3.1. Patient characteristics

A total of 152 patients (121 men; median age [interquartile range], 22 [19–30] years) were enrolled. Baseline characteristics are presented in Table 1. The median disease follow-up duration was 4.3 years. The sample comprised 15 (9.9%), 31 (20.4%), 69 (45.4%), 34 (22.4%), and 3 (2.0%) patients with disease-extent imaging scores of 0, 1, 2, 3, and 4, respectively.

**Table 1:** Baseline characteristics of enrolled patients.

Characteristics	All patients (n = 152)
Age (yr)	22 (19.30)
Male	121 (79.6%)
Smoking	
Never smoked	99 (65.1%)
Ex-smoker	26 (17.1%)
Current smoker	27 (17.8%)
Family history	10 (6.58%)
Disease duration (yr)	4.25 (2.18–6.46)
Age at diagnosis	
A1 (≤16 yr)	7 (4.61%)
A2 (17–40 yr)	128 (84.2%)
A3 (>40 yr)	17 (11.2%)
Location of disease	
L1 (terminal ileum)	38 (25.0%)
L3 (ileo-colon)	114 (75.0%)
L4 (upper disease)*	20 (13.2%)
Behavior of disease	
B1 (not structuring or penetrating)	84 (55.3%)
B2 (structuring)	33 (21.7%)
B3 (penetrating)	35 (23.0%)
Perianal disease	65 (42.8%)
Extraintestinal manifestation	13 (8.55%)
Disease-extent imaging score	
0	15 (9.9%)
1	31 (20.4%)
2	69 (45.4%)
3	34 (22.4%)
4	3 (2.0%)

Data are the number of patients (percentage) or median (interquartile range).

A = age, B = behavior, L = location.

* Classified at the time of diagnosis based on endoscopy and imaging findings.

### 3.2. Distribution and behavior of small bowel involvement

The proportions of patients with disease involvement in the jejunum, distal jejunum/proximal ileum, distal ileum, and terminal ileum were 2.0% (3/152), 30.3% (46/152), 82.2% (125/152), and 71.7% (109/152), respectively (Table 2). The most frequently affected site was the distal ileum. Strictures, fistulas, and abscesses tended to be more common in the distal ileum than in the other segments (Table 2). Montreal classification L4 (involvement of the jejunum or distal jejunum/proximal ileum) was observed in 30.3% (46/152) of the patients. Most patients with disease involvement in the distal jejunum/proximal ileum or distal ileum had multiple lesions.

**Table 2:** Distribution and characteristics of small bowel involvement on CT.

	Bowel wall thickening	Stricture	Fistula or abscess	Number of lesions	
				Single	Multiple*
Terminal ileum	109 (71.7%)	6 (3.9%)	9 (5.9%)	111 (73.0%)	N/A†
Distal ileum	125 (82.2%)	30 (19.7%)	27 (17.8%)	6 (3.9%)	119 (78.3%)
Proximal ileum or distal jejunum	46 (30.3%)	5 (3.2%)	1 (0.7%)	7 (4.6%)	39 (25.7%)
Jejunum	3 (2.0%)	0 (0%)	0 (0%)	1 (0.7%)	2 (1.3%)

Data are n (%, per all 152 patients).

* Two or more lesions regardless of the type (bowel wall thickening, stricture, fistula, or abscess).

† N/A, not assessed, the lesion of terminal ileum was regarded as one.

### 3.3. Risk factors associated with poor clinical outcomes in CD patients

**Figure 1.**
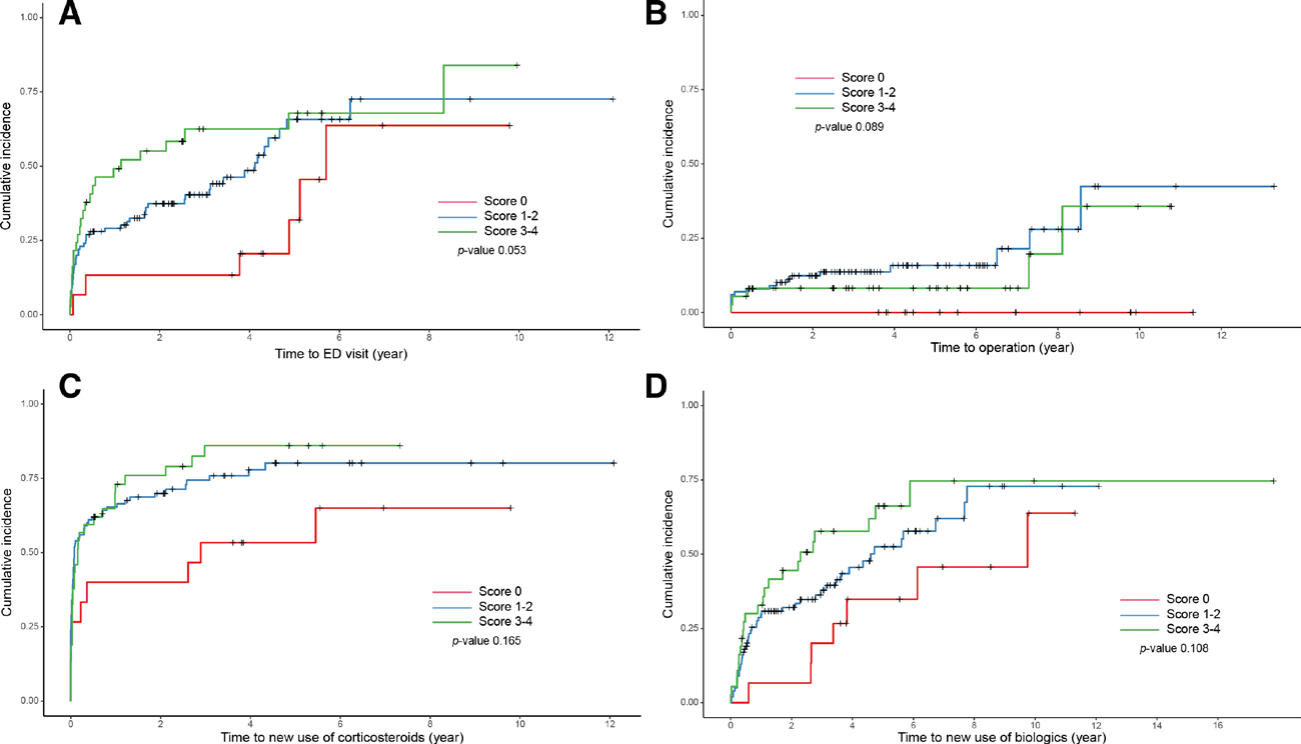
(A) Comparison of cumulative incidence curves for ED visit against the disease-extent imaging score. (B) Comparison of cumulative incidence curves for operation against the disease-extent imaging score. (C) Comparison of cumulative incidence curves for use of corticosteroids against the disease-extent imaging score. (D) Comparison of cumulative incidence curves for use of biologics against the disease-extent imaging score. ED = emergency department.

In Kaplan–Meier survival analysis, there was no significant difference in the clinical outcomes among the 3 groups divided according to the disease-extent imaging score (ED visit *P* = .053; operation *P* = .089; new use of corticosteroid *P* = .165; and new use of biologics *P* = .108) (Fig. 1A–D). Likewise, there was no significant difference in the clinical outcomes according to upper gastrointestinal involvement (i.e., L4 vs non-L4) (ED visit *P* = .099; operation *P* = .839; new use of corticosteroid *P* = .895; or new use of biologics *P* = .363) (Figure S1A–D, Supplemental Digital Content, http://links.lww.com/MD/J780, http://links.lww.com/MD/J782, http://links.lww.com/MD/J785, http://links.lww.com/MD/J787).

Table 3 shows the multivariable analyses of clinical outcomes. For ED visits, disease behavior (hazard ratio [HR], 2.127 [1.356–3.337], *P* = .001) and perianal disease (HR, 1.774 [1.136–2.771], *P* = .012) were significant risk factors. For operation, age 17 to 40 at diagnosis (HR, 0.229 [0.066–0.798], *P* = .021) and disease behavior (HR 8.216 [2.629–25.683], *P* < .001) were significant factors. For new use of corticosteroids, disease behavior (HR, 1.816 [1.249–2.642], *P* = .002) and ileo-colon disease (L3 vs L1; HR, 1.648 [1.050–2.586], *P* = .030) were significant risk factors. For new use of biologics, disease behavior (HR, 2.352 [1.492–3.708], *P* < .001) was the only risk factor. Taken together, the results show that disease behavior was the only risk factor associated with all 4 clinical outcomes after adjusting for other factors.

**Table 3:** Multivariable analysis for risk factors associated with clinical outcomes.

Variates	Emergency department visit		Operation		New use of corticosteroid		New use of biologics	
	HR (95% CI)	*P* value	HR (95% CI)	*P* value	HR (95% CI)	*P* value	HR (95% CI)	*P* value
Age								
A1 (<17)	Reference		Reference				Reference	
A2 (17–40)	0.463 (0.195–1.097)	.080	0.229 (0.066–0.798)	**.021**			0.549 (0.217–1.390)	.206
A3 (>40)	0.360 (0.112–1.164)	.088	0.104 (0.011–1.012)	.051			0.307 (0.087–1.087)	.067
Behavior (B2/B3)	2.127 (1.356–3.337)	**.001**	8.216 (2.629–25.683)	**<.001**	1.816 (1.249–2.642)	**.002**	2.352 (1.492–3.708)	**<.001**
Location (L3)			0.477 (0.192–1.182)	.110	1.648 (1.050–2.586)	**.030**		
Perianal disease	1.774 (1.136–2.771)	**.012**						
Disease-extent imaging score								
0	Reference				Reference		Reference	
1–2	1.313 (0.526–3.278)	.559			1.528 (0.739–3.157)	.252	1.061 (0.445–2.534)	.893
3–4	1.798 (0.681–4.745)	.236			1.368 (0.623–3.001)	.435	1.540 (0.627–3.782)	.347
Smoking								
Never	Reference							
Ex-smoker	0.601 (0.280–1.292)	.192						
Current	1.152 (0.636–2.089)	.641						

A = age, B = behavior, EIM = extraintestinal manifestation, L = location.

### 3.4. Ad-hoc analysis using the behavior-weighted imaging score

Figure S2, Supplemental Digital Content, http://links.lww.com/MD/J791 shows the distribution of patients by behavior-weighted imaging score. We observed 23 (15.1%), 72 (47.4%), and 57 (37.5%) patients in groups 0–1, 2–4, and ≥5, respectively. In Kaplan–Meier survival analysis, we observed significant differences among the 3 groups for ED visit (*P* = .001) and new use of biologics (*P* = .030). We found no significant difference in operations (*P* = .239) and new use of corticosteroids (*P* = .228) (Figure S3A–D, Supplemental Digital Content, http://links.lww.com/MD/J793, http://links.lww.com/MD/J796, http://links.lww.com/MD/J798, http://links.lww.com/MD/J800). In the multivariable analysis, a behavior-weighted imaging score of 5 to 40 (HR, 2.488 [1.230–5.033], *P* = .011) and perianal disease (HR, 1.678 [1.070–2.632], *P* = .024) were significant risk factors for ED visits. For operation, age 17 to 40 at diagnosis (HR, 0.264 [0.076–0.919], *P* = .036) and ileo-colon disease (L3 vs L1; HR, 0.303 [0.124–0.743], *P* < .009) were significant factors. We observed no significant risk factors for new use of corticosteroids. For new use of biologics, a behavior-weighted imaging score of 5 to 40 (HR, 2.108 [1.069–4.156], *P* = .031) was the only significant risk factor (Table S1, Supplemental Digital Content, http://links.lww.com/MD/J803).

### 3.5. Inter-observer agreement

Agreement on fistula or abscess was almost perfect (kappa value = 1.0 for most segments). The degree of agreement on bowel-wall thickening and stricture varied according to the involved segments (kappa value = 0.690–0.936) (Table S2, Supplemental Digital Content, http://links.lww.com/MD/J804).

## 4. Discussion

In this study, we evaluated the distribution of small bowel involvement in CD and analyzed the clinical outcomes according to the disease extent, location (L4 vs non-L4 disease), and behavior (structuring or fistulizing nature). Our results suggest that disease behavior at initial diagnosis is the most significant risk factor for clinical outcomes in patients with CD.

Before conducting our pre-planned analysis based on the disease-extent imaging score and location, we anticipated that disease extent or L4 involvement would be associated with the clinical outcomes. Contrary to our hypotheses, we observed no statistically significant differences in clinical outcomes by disease extent or L4 involvement, although we found some tendency for patients with a greater disease extent (i.e., a higher disease-extent imaging score) to have a higher incidence of poor clinical outcomes. Based on our observation that initial disease behavior was the only risk factor associated with all 4 clinical outcomes, we performed an ad-hoc analysis on the behavior-weighted imaging score, and observed that the score was significantly associated with the time to ED visits and the use of biologics. Since calculating the behavior-weighted imaging score would be burdensome in daily practice, clinicians may alternatively use the disease behavior at initial diagnosis to predict clinical outcomes. The HR of patients with high behavior-weighted imaging score (≥5) (2.488 for ED visits and 2.108 for new use of biologics) were similar to those of patients with B2/B3 behavior in our initial analysis (2.127 for ED visit, and 2.352 for new use of biologics).

The Montreal classification of each patient was determined at initial diagnosis based on endoscopy and imaging modalities. Twenty (13.2%) patients were classified as having L4 disease at initial diagnosis, in contrast to 46 (30.3%) patients in the retrospective CT image review. This may be because some official CT reports lacked detailed descriptions regarding the segment of the small bowel involved, and we took a conservative stance by classifying those cases as non-L4 disease.

The association of L4 disease with prognosis remains uncertain in patients with CD, with discrepant results in previous studies. While a few studies have reported that L4 disease is associated with a poorer prognosis, some showed results that suggested otherwise.^[9–13]^ Our results did not show a significant association between L4 disease and clinical outcomes. This may be because most of the strictures (B2) or fistula/abscesses (B3), which became the most decisive factor for the clinical outcomes, occurred mainly in the distal ileum. A previous study showed similar results, in which most fistulae (95%) originated from the ileum.^[20]^

In the retrospective CT image review, we observed that many patients (69.7%) had small bowel involvement in multiple segments. Our disease-extent imaging score was intended to reflect the extent of small bowel involvement, where a higher score indicated a greater number of involved small bowel segments. The disease-extent imaging score showed a tendency to be associated with clinical outcomes, but not strongly enough to be statistically significant. This may be attributable to insufficient statistical power in detecting the difference or due to the predominant effect of stricture or fistula in the distal ileum.

Among the 4 clinical outcomes, operation showed a pattern distinct from that of the other outcomes. More surgeries were performed in patients with a disease-extent imaging score of 1 or 2 than in those with a score of 3 or 4. A possible explanation for this is that surgical resection is known to be as effective as medical treatment for patients with small bowel involvement localized to 1 or 2 segments.^[21–23]^ In contrast, if multiple segments are involved, surgical resection can lead to complications, such as short-bowel syndrome. Therefore, it would have been easier for clinicians to decide to operate the patient with a score 1 or 2 than in the patients with a score of 3 or 4.

A major strength of this study is the use of data collected prospectively in a cohort, which may have reduced the biases associated with retrospective data collection. Except for those from the retrospective CT image review, all data were prospectively collected, including the clinical outcomes. We tried to minimize the biases associated with retrospective reviews by blinding the radiologists to any relevant clinical information.

This study has several limitations. First, we did not consider colon involvement, and the extent and severity of colonic involvement may have affected our analyses. Second, since the small bowel was assessed only via CT, we may have missed the small-bowel involvements that could be identified using other modalities such as enteroscopy or capsule endoscopy. Moreover, although the inter-observer agreement between the 2 radiologists was nearly perfect for fistula or abscess, we found some variability in bowel wall thickening. To overcome this limitation, we considered the presence of bowel wall thickening only if both radiologists concurred.

In conclusion, according to a review of CTs acquired at the initial diagnosis, approximately one-third of patients had L4 disease, and two-thirds showed multi-segmental involvement. However, disease extent and distribution in the small bowel did not show a significant correlation with clinical outcomes. Instead, initial disease behavior was the strongest predictor of clinical outcomes. Therefore, initial studies for the diagnosis of CD should emphasize the detection of structuring or fistulizing disease.

## Author contributions

**Conceptualization:** Yoon Jin Lee, Hyuk Yoon.

**Formal analysis:** Jin Park, Hae Young Kim.

**Investigation:** Cheol Min Shin, Young Soo Park, Nayoung Kim, Dong Ho Lee.

**Writing – original draft:** Jin Park, Hae Young Kim.

**Writing – review & editing:** Yoon Jin Lee, Hyuk Yoon.

## Supplementary Material
