# Supplementary figures and images for: Distribution of small bowel involvement and its association with clinical outcomes in patients with Crohn’s disease
Source: Medicine (Baltimore). 2023 Oct 6;102(40):e35040. doi: 10.1097/MD.0000000000035040 (PMC10553183; doi:10.1097/MD.0000000000035040)

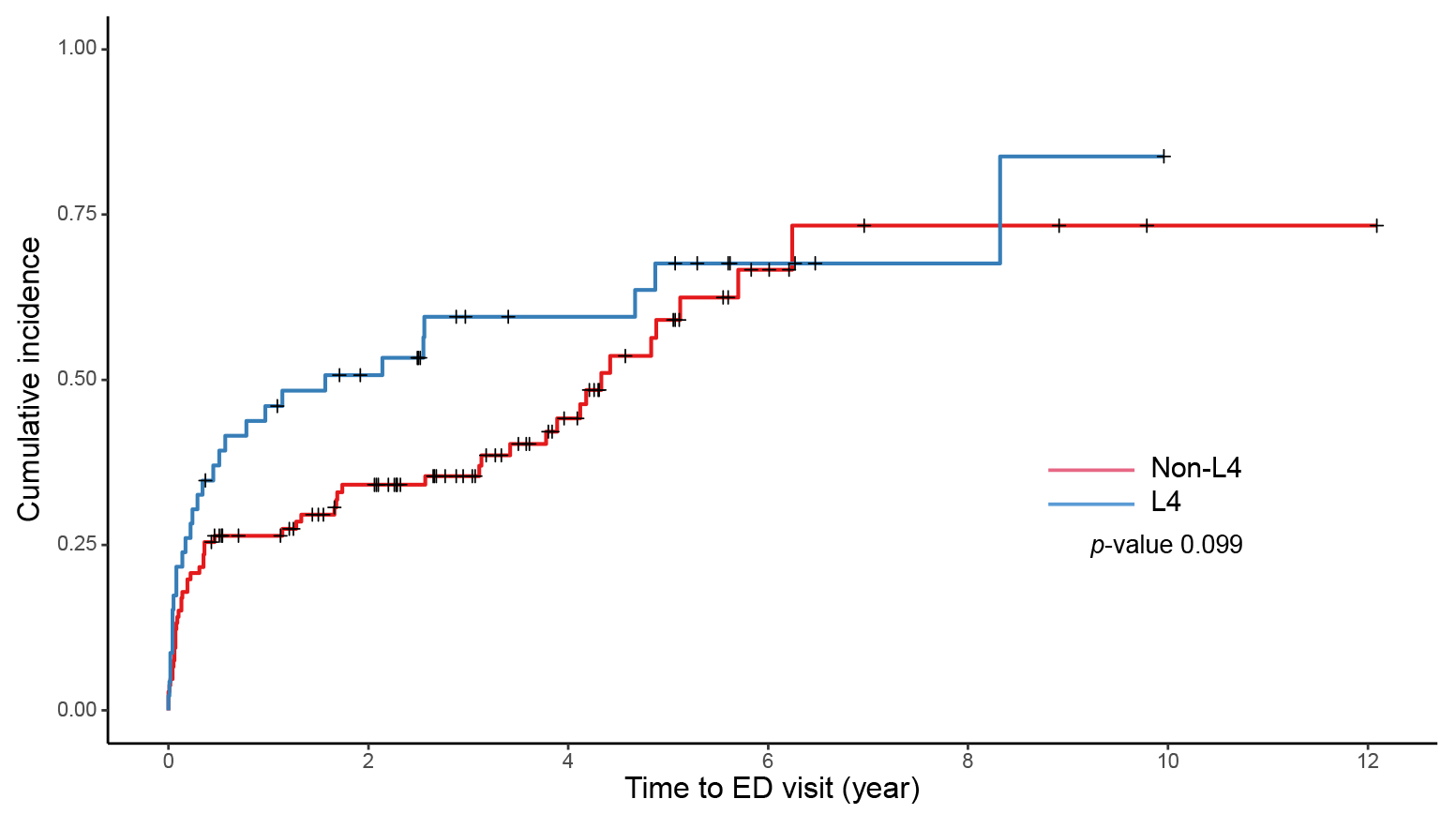

Supplement: Supplementary file 1 [file medi-102-e35040-s001.tif]

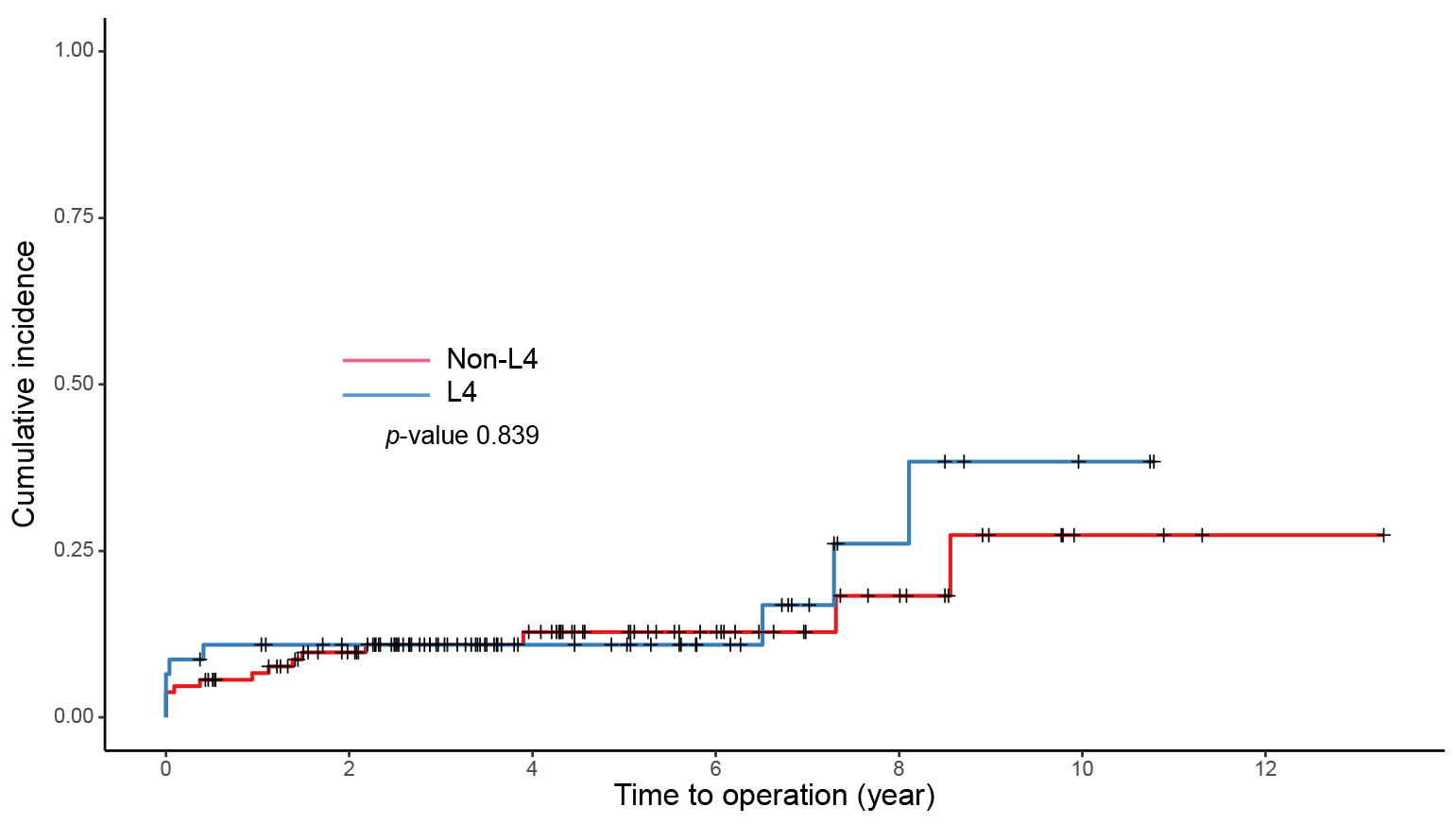

Supplement: Supplementary file 2 [file medi-102-e35040-s002.tif]

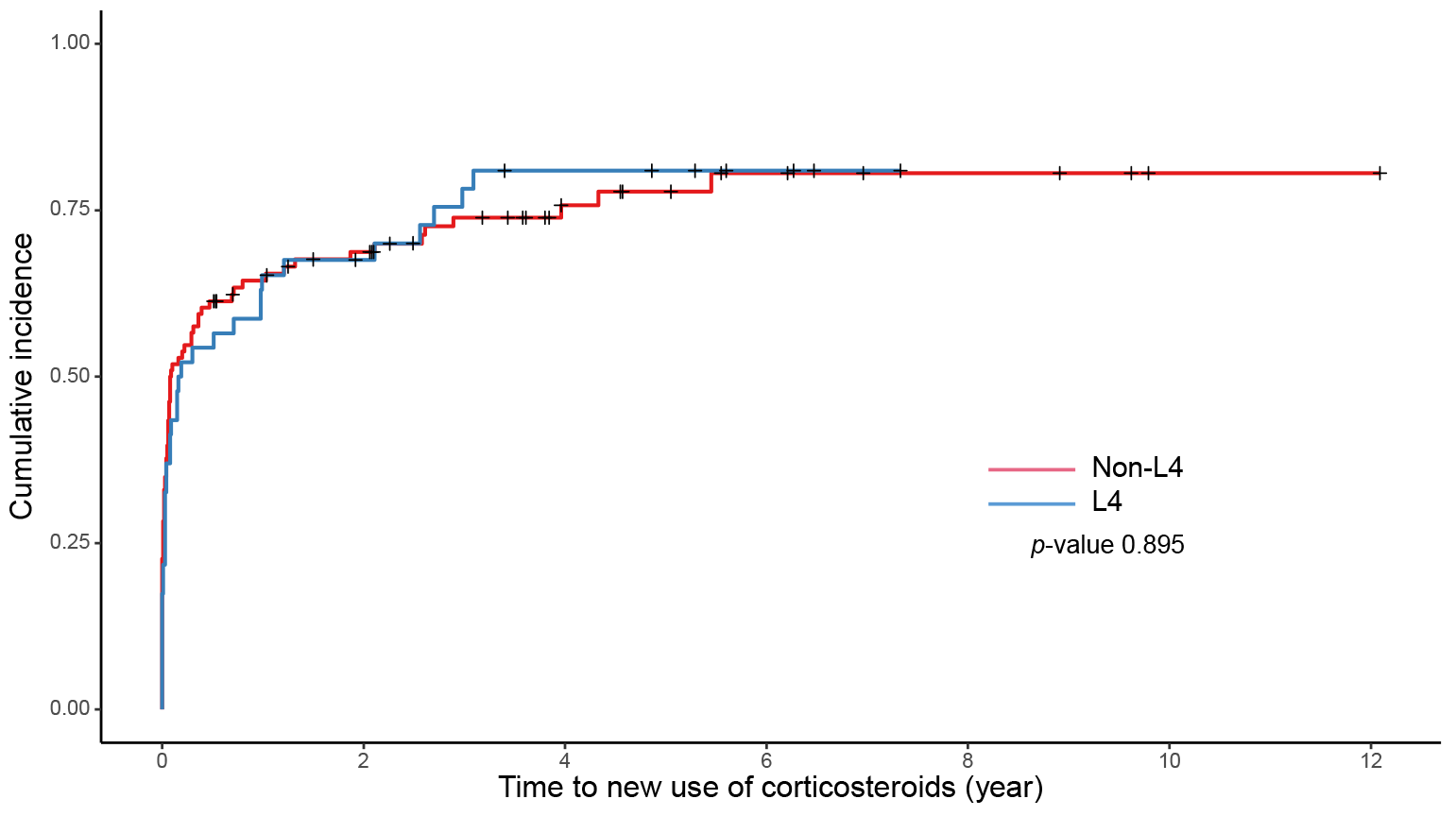

Supplement: Supplementary file 3 [file medi-102-e35040-s003.tif]

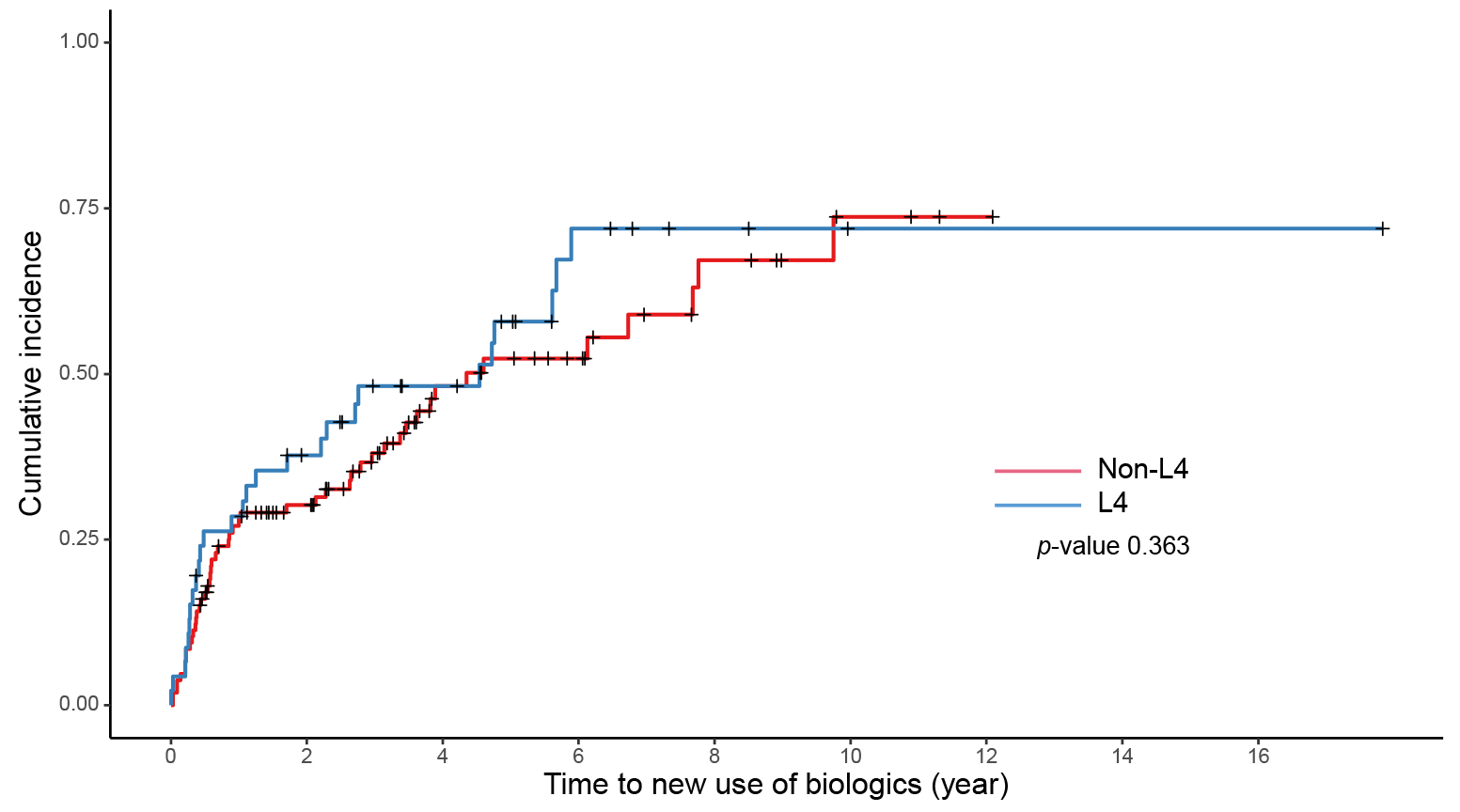

Supplement: Supplementary file 4 [file medi-102-e35040-s004.tif]

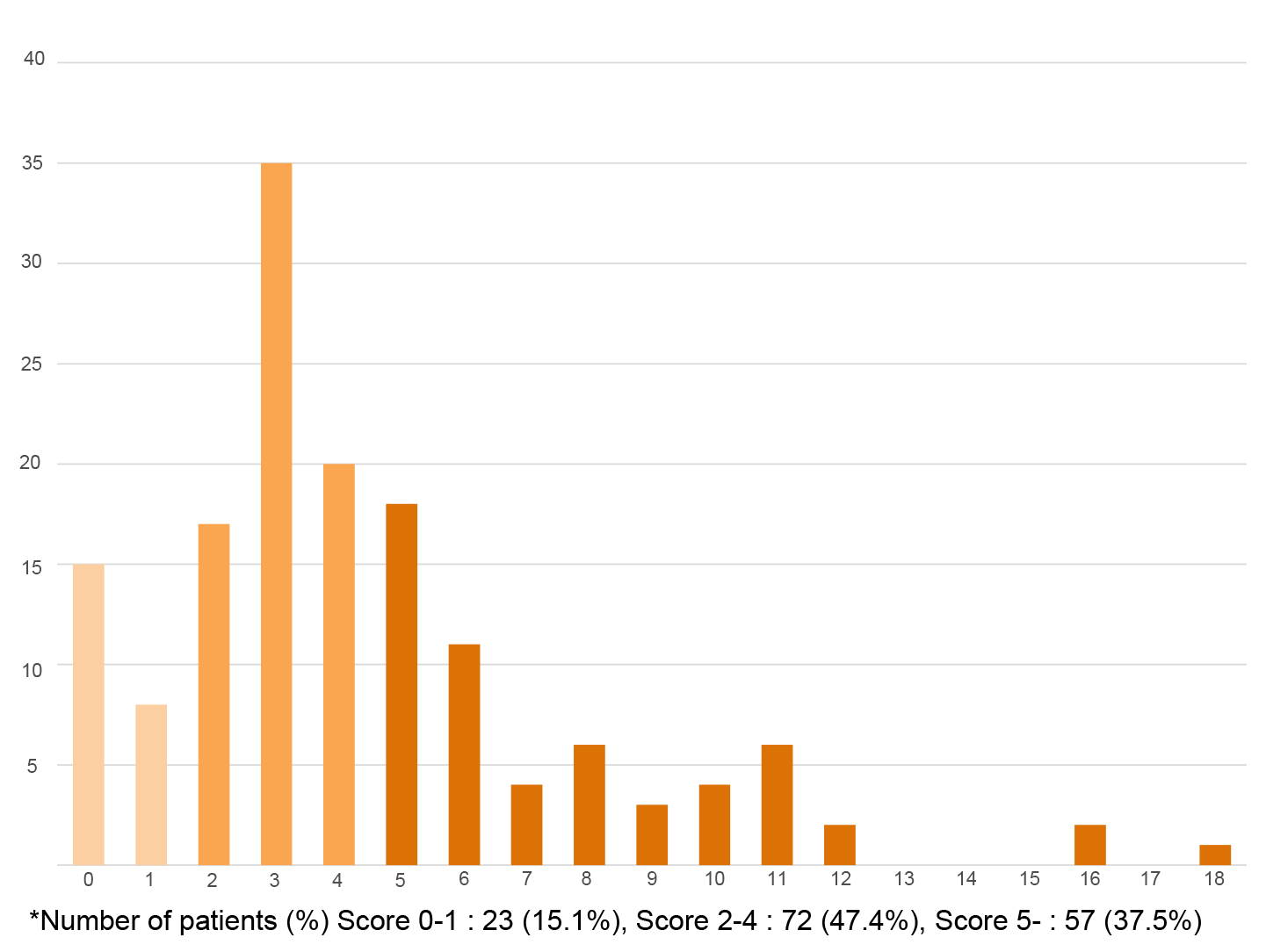

Supplement: Supplementary file 5 [file medi-102-e35040-s005.tif]

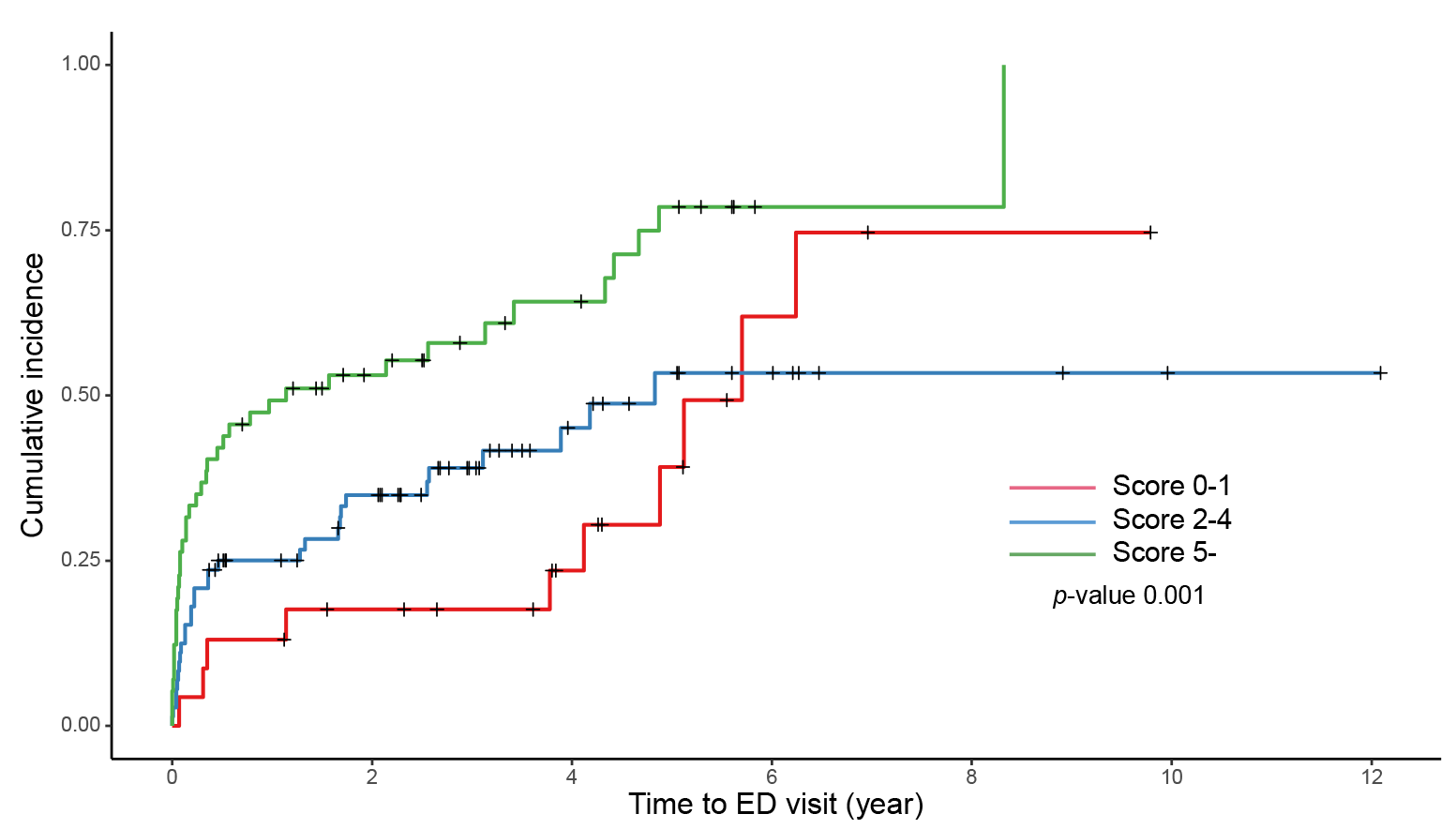

Supplement: Supplementary file 6 [file medi-102-e35040-s006.tif]

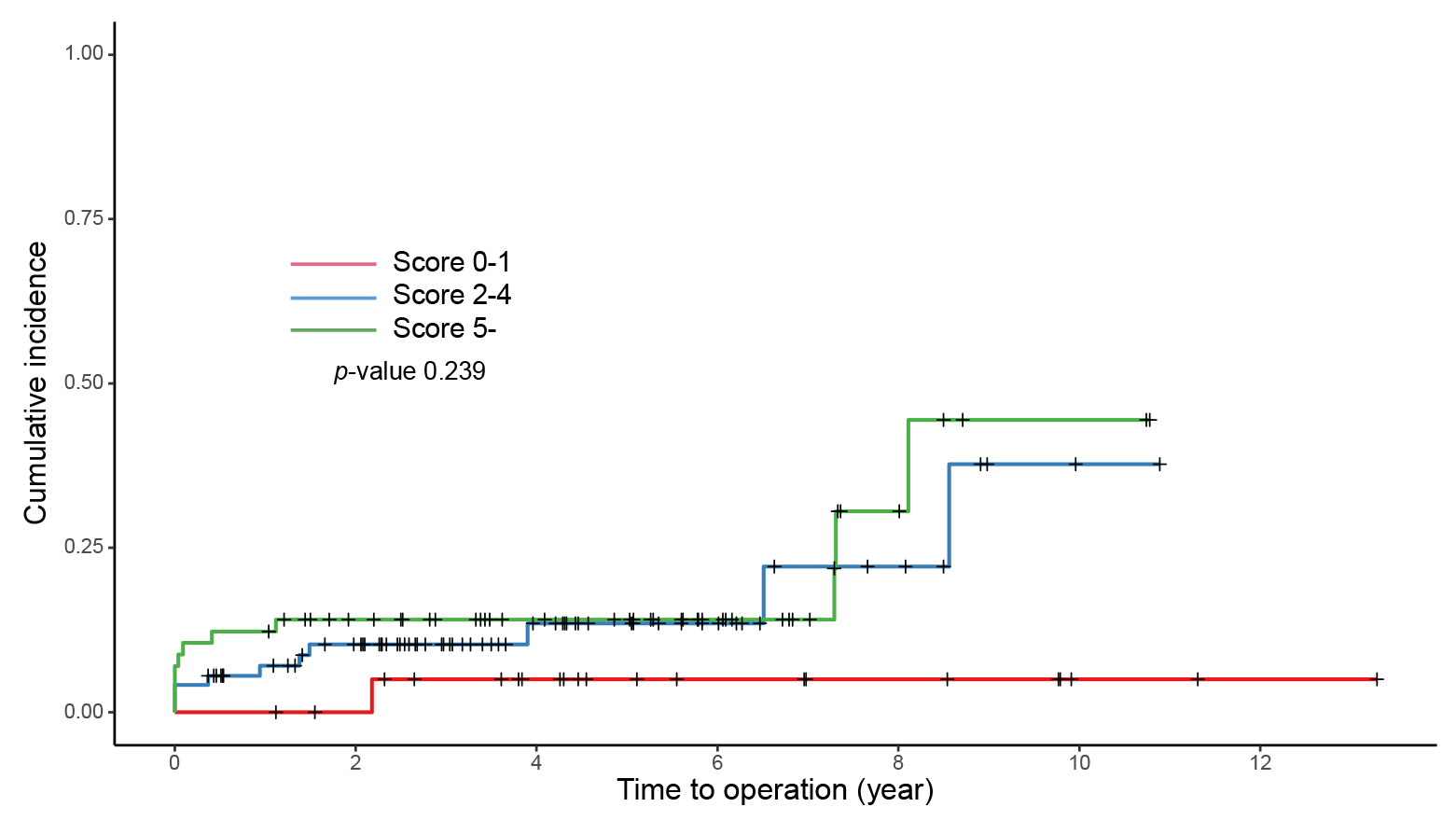

Supplement: Supplementary file 7 [file medi-102-e35040-s007.tif]

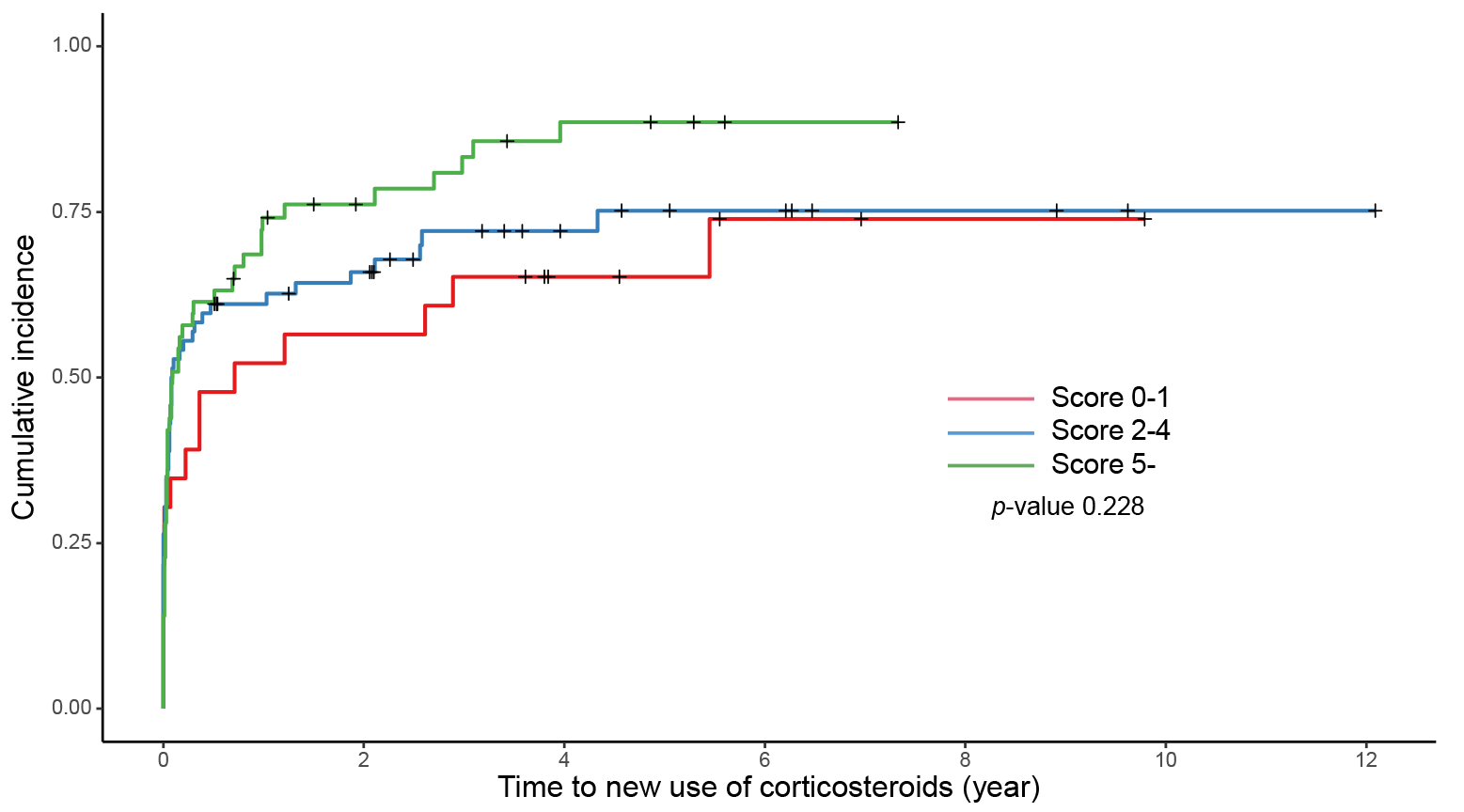

Supplement: Supplementary file 8 [file medi-102-e35040-s008.tif]

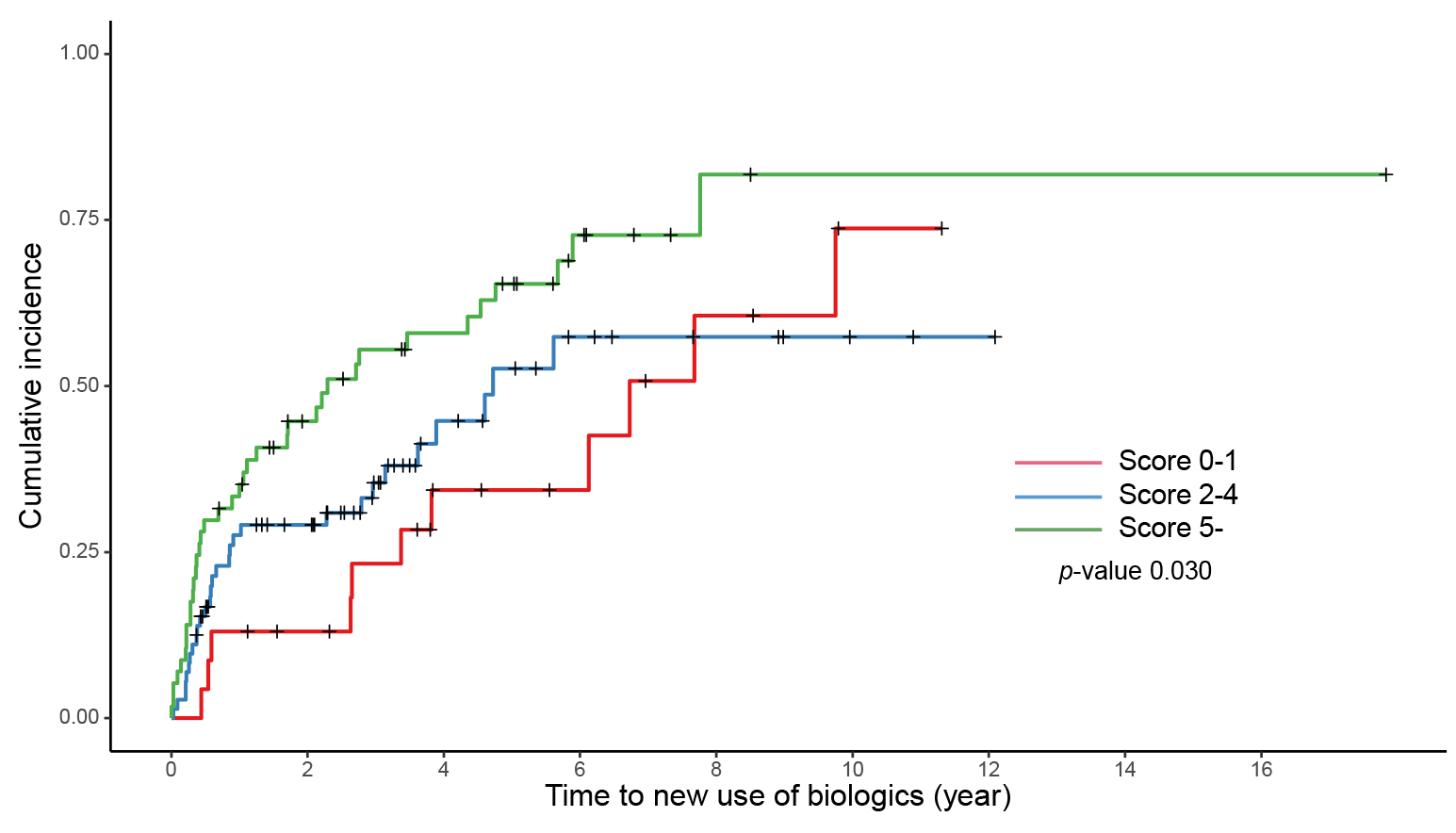

Supplement: Supplementary file 9 [file medi-102-e35040-s009.tif]
