# Supplementary material for: Distribution of small bowel involvement and its association with clinical outcomes in patients with Crohn’s disease
Source: Medicine (Baltimore). 2023 Oct 6;102(40):e35040. doi: 10.1097/MD.0000000000035040 (PMC10553183; doi:10.1097/MD.0000000000035040)
Supplement: Supplementary file 10 [file medi-102-e35040-s010.docx]

**Supplementary Table 1.** Multivariable Analysis for Risk Factors Associated with Clinical Outcomes, Including the Variable Behavior-weighted Imaging Score.

| Variates | Emergency department visit | | Operation | | New use of corticosteroid | | New use of biologics | |
| --- | --- | --- | --- | --- | --- | --- | --- | --- |
|  | HR (95% CI) | p-value | HR (95% CI) | p-value | HR (95% CI) | p-value | HR (95% CI) | p-value |
| Age |  |  |  |  |  |  |  |  |
| A1 (< 17) | Reference |  | Reference |  |  |  | Reference |  |
| A2 (17-40) | 0.505 (0.214-1.195) | 0.120 | 0.264 (0.076-0.919) | **0.036** |  |  | 0.640 (0.254-1.610) | 0.343 |
| A3 (> 40) | 0.455 (0.140-1.479) | 0.190 | 0.104 (0.011-1.025) | 0.053 |  |  | 0.360 (0.098-1.323) | 0.124 |
| Perianal disease | 1.678 (1.070-2.632) | **0.024** |  |  |  |  |  |  |
| Location (L3) |  | | 0.303 (0.124-0.743) | **0.009** | 1.490 (0.954-2.328) | **0.080** |  |  |
| Behavior-weighted imaging score |  |  |  |  |  |  |  |  |
| 0-1 | Reference |  |  |  |  |  | Reference |  |
| 2-4 | 1.316 (0.641-2.701) | 0.454 |  |  |  |  | 1.256 (0.624-2.528) | 0.522 |
| 5- | 2.488 (1.230-5.033) | **0.011** |  |  |  |  | 2.108 (1.069-4.156) | **0.031** |
| Smoking |  |  |  |  |  |  |  |  |
| Never | Reference |  |  |  |  |  |  |  |
| Ex-smoker | 0.546 (0.259-1.155) | 0.113 |  |  |  |  |  |  |
| Current | 1.088 (0.611-1.939) | 0.774 |  |  |  |  |  |  |

A = age, B = behavior, L = location, EIM = extraintestinal manifestation.
