# Supplementary material for: Distribution of small bowel involvement and its association with clinical outcomes in patients with Crohn’s disease
Source: Medicine (Baltimore). 2023 Oct 6;102(40):e35040. doi: 10.1097/MD.0000000000035040 (PMC10553183; doi:10.1097/MD.0000000000035040)
Supplement: Supplementary file 11 [file medi-102-e35040-s011.docx]

**Supplementary Table 2.** Inter-observer Agreement in Retrospective CT Image Review.

|  | Bowel wall thickening | Stricture | Fistula | Abscess |
| --- | --- | --- | --- | --- |
| Terminal ileum | 0.813 (0.707-0.920) | 0.690 (0.434-0.946) | 1 (1-1) | 1 (1-1) |
| Distal ileum | 0.936 (0.863-1) | 0.812 (0.700-0.924) | 0.945 (0.869-1) | 1 (1-1) |
| Proximal ileum or distal jejunum | 0.909 (0.838-0.981) | 0.759 (0.494-1) | 1 (1-1) | 1 (1-1) |
| Jejunum | 0.743 (0.398-1) | NaN^*^ | NaN^*^ | NaN^*^ |

Data within parentheses represent Cohen’s kappa coefficients with 95% confidence interval.

^*^NaN: Computation was not possible because all patients received the same rating from both radiologists.
